# Long-term outcomes for women after obstetric fistula repair in Lilongwe, Malawi: a qualitative study

**DOI:** 10.1186/s12884-015-0755-1

**Published:** 2016-01-05

**Authors:** Laura B. Drew, Jeffrey P. Wilkinson, William Nundwe, Margaret Moyo, Ronald Mataya, Mwawi Mwale, Jennifer H. Tang

**Affiliations:** UNC Project-Malawi, Lilongwe, Malawi; Baylor College of Medicine, Houston, TX USA; Freedom from Fistula Care Centre, Lilongwe, Malawi; Malawi College of Medicine Department of OB-GYN, Blantyre, Malawi; Loma Linda University School of Public Health, Loma Linda, CA USA; Lilongwe District Health Office, Lilongwe, Malawi; UNC Department of OB-GYN, Chapel Hill, NC USA

## Abstract

**Background:**

Obstetric fistula affects a woman’s life physically, psychosocially, and economically. Although surgery can repair the physical damage of fistula, the devastating consequences that affect a woman’s quality of life may persist when she reintegrates into her community. This qualitative study assessed long-term outcomes among women who underwent obstetric fistula repair in Malawi. We explored three domains: overall quality of life before and after repair, fertility and pregnancy outcomes after repair, and understanding of fistula.

**Methods:**

In-depth interviews were conducted in Chichewa with 20 women from seven districts across Central Malawi. All women were interviewed 1 to 2 years after surgical repair for obstetric fistula at the Fistula Care Centre in Lilongwe, Malawi. Interviews were independently coded and analyzed using content analysis.

**Results:**

About half of women were married and nine of 20 women reported some degree of urinary incontinence. With the exception of relationship challenges, women’s concerns before and after repair were different. Additionally, repair had resolved many of the concerns women had before repair. However, challenges, both directly and indirectly related to fistula, persisted. Improvements in quality of life at the individual level included feelings of freedom, confidence and personal growth, and improved income-earning ability. Interpersonal quality of life improvements included improved relationships with family and friends, reduced stigma, and increased participation with their communities. Nearly half of women desired future pregnancies, but many were uncertain about their ability to bear children and feared additional pregnancies could cause fistula recurrence. Most women were well informed about fistula development but myths about witchcraft and fear of delivery were present. Nearly all women would recommend fistula repair to other women, and many were advocates in their communities.

**Conclusions:**

Nearly all women believed their quality of life had improved at the individual and interpersonal levels since fistula repair, even among women who continued to have urinary incontinence. Contrary to other studies, women reported they were welcomed back by their communities and had limited challenges when reintegrating. Despite the overall improvements in quality of life, many continued to have relationship problems and were concerned about future fertility. These issues need to be further explored in other studies.

## Background

Obstetric fistula is a devastating condition that can develop when women experience prolonged obstructed labor. During this childbirth complication, the soft tissues of the pelvis become necrotic due to lack of blood supply, causing an abnormal opening to form between the vagina and the bladder and/or rectum [1]. This damage leads to chronic incontinence of urine and/or feces. Without treatment, obstructed labor is estimated to contribute to 8 % of maternal deaths worldwide [2]. In developing countries, obstetric fistula are estimated to occur in 2 % of obstructed labors [3]. The World Health Organization estimates that more than two million girls and women currently live with fistula [4]. In Malawi, the prevalence of vesicovaginal fistula is believed to be 1.6 in 1,000 women [5].

Obstetric fistula is particularly devastating because it is both physically and socially disabling to women [6–11]. In addition to causing chronic incontinence, obstetric fistula often affects the health and well-being of women socially, economically, and psychologically [7, 8, 10–12]. In 1996, Arrowsmith and colleagues presented the “Obstructed Labor Injury Complex” to describe the multitude of physical and social injuries caused by obstetric fistula [13]. Significant consequences of obstetric fistula that are described in the “Obstructed Labor Injury Complex” include the following: secondary infertility, chronic skin irritation due to incontinence, stigmatization due to offensive odor, isolation and loss of social support, divorce or separation, worsening poverty, worsening malnutrition, as well as suffering, illness, and premature death.

Because obstetric fistula can cause life-long disabilities [8], it affects a woman’s productivity, as well as the productivity of her household and community [10]. Success rates of obstetric fistula repair are high and have been reported in the literature [14]; however, there are limited data on long-term outcomes following fistula repair, particularly the quality of life among patients who had fistula surgery [15]. Previous research in Malawi found women’s lived experiences with obstetric fistula are varied, specifically their continued participation in marriage, their community, and childbearing [16]. Additional research by Yeakey et al. focused on the need to understand Malawian women’s perceptions about successful fistula repair and how it affects their families and home environments [17]. Despite the study’s findings that successful fistula repair among Malawian women can contribute to positive outcomes for the women, their families, and communities [17], little is known about the long-term outcomes and quality of life after fistula repair in Malawi. Furthermore, successful surgical treatment alone does not address the totality of obstetric fistula consequences: even with the physical injury repaired, women who have had fistula may face ongoing depression, ostracization from their families or communities, reduced opportunities for income, and other negative outcomes.

Identifying whether or not psychosocial, economic, and reproductive health challenges persist after fistula repair is necessary to assess the long-term quality of life for women after fistula repair. This will also aid intervention efforts that address the non-physical consequences of obstetric fistula. Therefore, we conducted this study to better understand the long-term quality of life and outcomes among women who underwent fistula repair at the Fistula Care Centre in Lilongwe, Malawi.

## Methods

### Study design

This study was a qualitative study of 20 Malawian women who had previously undergone obstetric fistula repair. All study participants underwent informed consent by trained study staff (WN) using an IRB-approved informed consent form in Chichewa. The study was approved by both the UNC Biomedical Institutional Review Board and the Malawi National Health Sciences Research Committee. After completing the study, participants received approximately $5 in Malawian currency as remuneration.

Participants were selected via purposive sampling. Eligibility criteria included: 1) history of fistula repair surgery at the Fistula Care Centre in Lilongwe, Malawi within the past 1–2 years, 2) age 18 years or older, 3) willingness to have their interview recorded, 4) residence in one of the 9 central Malawian districts, and 5) the ability to locate participants and/or contact them via working phone numbers. As a part of their routine care, women who undergo repair at the Fistula Care Centre are scheduled to complete follow-up questionnaires at one month and three months post repair. Although all of the women we interviewed had previously returned for follow-up, many women have been unable to return due to financial or personal constraints. For this reason and to ensure the women felt comfortable during the interview, we conducted interviews within participants’ homes or at local health centers, whichever they preferred. We estimated a sample size of approximately 20 women was necessary to reach data saturation for our predetermined themes. These themes were based on our previous interactions with patients at the Fistula Care Centre and our research on women’s experiences before and after fistula repair.

### Data collection and analysis

From July to September 2013, a trained interviewer (WN) conducted in-depth interviews with the participants in Chichewa, which were designed to take 30–60 min to complete. Basic demographic information was collected at the beginning of the interview. This data was recorded by hand and later entered into an Excel spreadsheet. Eighteen participants were interviewed in their homes and 2 participants were interviewed at local health centers. Interviews were audiotaped, transcribed, translated into English, and typed verbatim into Microsoft Word by the interviewer (WN), a Malawian research associate fluent in Chichewa and English. Two researchers (LBD, WN) then independently hand-coded the interviews using content analysis in Microsoft Word to identify major themes. During analysis, codes were developed and refined, and themes were extracted to identify and explain the data’s core meanings. During the course of the study, modifications were made to the interview guide as new themes and ideas requiring further investigation were revealed. LBD, WN, and JHT held weekly meetings to discuss the interviews/coding and make appropriate modifications to the study guide. Our interview guide focused on three major domains: 1) Overall quality of life, 2) Fertility and pregnancy after repair, and 3) Understanding of fistula. The data coding by WN and LBD were triangulated. The comparisons consistently supported data validity between investigators. At the end of data collection, an Excel spreadsheet was created to identify frequency of code(s) usage among participant(s). Of note, participants who were found during their interviews to have medical or psychological needs and required medical attention were given follow-up appointments at the Fistula Care Centre for evaluation by medical staff.

## Results

### Description of participants

We contacted and recruited 20 women to be in this study, all of whom agreed to participate. The 20 women were between 19 and 76 years of age (Table 1). The women were from seven districts in Central Malawi: most were from Dowa, Lilongwe, and Nkhotakota. The majority had attended some primary school (60 %); nevertheless, a large number of women had never attended school (30 %). When asked about religion, more than half (55 %) identified with religions other than Christianity or Islam, the two most common religions in Malawi. At the time of the interview, nearly all women were working (90 %). Nearly all women were married immediately prior to fistula development (80 %); however, only about half were married at the time of the interview (55 %). The number of living children women had varied greatly, but most developed their fistula between 15 and 19 years of age (40 %). Almost half (45 %) continued to have residual urinary incontinence at the time of the interview; however, in-depth interviews revealed nearly all women felt the quality of their lives had improved despite the incontinence. During the interviews, many sub-themes emerged, which revealed other psychosocial issues experienced after fistula repair (Table 2).

**Table 1:** Characteristics of study participants

Characteristic	*n* (%)
# of study participants	20
Age	
<20	1 (5 %)
20–29	5 (25 %)
30–39	8 (40 %)
40–49	1 (5 %)
≥50	4 (20 %)
Don’t know	1 (5 %)
District	
Kasungu	2 (10 %)
Ntcheu	1 (5 %)
Nkhotakota	4 (20 %)
Dowa	6 (30 %)
Mchinji	1 (5 %)
Dedza	1 (5 %)
Lilongwe	5 (25 %)
Religion	
Protestant	1 (5 %)
Catholic	7 (35 %)
Muslim	1 (5 %)
Other	11 (55 %)
Education	
Never attended school	6 (30 %)
Some primary school	12 (60 %)
Finished primary school or more	2 (10 %)
Occupation	
Farmer	9 (45 %)
Housewife/Caretaker	1 (5 %)
Business owner	7 (35 %)
House worker	1 (5 %)
No occupation	2 (10 %)
Relationship status	
Married	11 (55 %)
Single	2 (10 %)
In a relationship but not married	1 (5 %)
Married but separated	3 (15 %)
Divorced	2 (10 %)
Widowed	1 (5 %)
Number of living children	
0	9 (45 %)
1	3 (15 %)
2	3 (15 %)
3	2 (10 %)
4	2 (10 %)
5	1 (5 %)
Age when fistula developed	
<15	1 (5 %)
15–19	8 (40 %)
20–24	4 (20 %)
25–29	4 (20 %)
30–34	1 (5 %)
Don’t know	2 (10 %)
Residual urinary incontinence	
Cured, no incontinence	11 (55 %)
Incontinence with cough, strain or exertion	1 (5 %)
Incontinent on walking	4 (20 %)
Incontinent on walking, sitting, and/or lying but still voiding some urine	2 (10 %)
Incontinent on walking, sitting, and/or lying but not voiding any urine	2 (10 %)

**Table 2:** Domains, categories, themes, and sub-themes from interviews with Malawian women 1–2 years post fistula repair surgery

Domain	Category/level	Theme	Sub-theme
Overall Quality of Life (QOL)	Greatest concerns before surgery	Death during surgery	
		Irreparable Fistula	
		Marital discord	(1) Partner abandonment
			(2) Polygamy
			(3) Fistula affected sexual intercourse
	Greatest concerns after surgery	Financial challenges	
		Additional surgery	
		Reproductive health and fertility	(1) Desire to have children
		Relationship challenges	(1) Unsupportive relationships
			(2) Fear of being unmarried
			(3) Challenges with sexual intercourse
	Individual Level	Compared to before surgery, QOL had improved	(1) QOL after surgery is similar to QOL before fistula developed
	Interpersonal Level	Relationships with family and friends before fistula repair	(1) Stigma from family, friends, and community
		Relationships with family and friends after fistula repair	(1) Few with ongoing stigma after repair
		Participation in social activities before surgery	(1) Isolation because of urine leakage
			(2) Inability to work or attend activities
		Participation in social activities after surgery	(1) Reintegration back into social and work activities
			(2) Holding positions within groups
Fertility and pregnancy after repair		Fertility desires and pregnancy outcomes	(1) Fear of fistula recurrence from future pregnancy
			(2) Confusion about ability to bear children
			(3) Consequences of not being able to have children
		Family planning use	
Understanding of fistula		Knowledge of fistula	(1) Delay seeking hospital care contributes to fistula development
			(2) Understanding prolonged labor as a cause of fistula but poor understanding of cause of prolonged labor
			(3) Cultural beliefs
		Fistula advocacy to others	(1) Assisted women with fistula to seek repair
			(2) Advocating for fistula prevention in communities
		Suggestions for helping women/with fistula	

### Overall quality of life

To better assess long-term outcomes after repair, women were asked about their greatest concerns before and after fistula surgery. For many women, relationship challenges were among their greatest concerns both before and after repair. Women were also asked about their overall quality of life, which we grouped into two levels: individual and interpersonal. Themes and sub-themes within these levels further described long-term outcomes and quality of life after repair.

#### Greatest concerns before surgery

Prior to fistula repair, women expressed a number of concerns that overwhelmed their lives. The concerns that were most commonly mentioned as their “greatest” concerns were: 1) death during surgery, 2) having an irreparable fistula, and 3) marital discord. Prior to repair, women were also concerned about urinary leakage, difficulties conceiving, their inability to marry, and negative thoughts, although these concerns were described less frequently.

More than a third of women stated one of their greatest concerns was that they would not survive fistula repair surgery (*n* = 7, 35 %). Fear stemmed from apprehension to undergo surgery, either because this was a woman’s first operation or because she had negative outcomes with previous surgeries.

*“You know, I came from another operation the time I was delivering my baby and it was a very painful time. So I thought that if I went for the fistula surgery I would die. I was crying all day because of that.”* (21-year-old woman with 3 living children)

Prior to repair, women were also very concerned that they would live their entire life with fistula. Nearly half doubted their condition could be repaired (*n* = 9, 45 %). Before visiting the Fistula Care Centre, many did not know that repair was possible and many believed their fistula was too complicated to repair.

*“I was not sure if I would get back to the way I was at first. I thought the problem was not repairable. I used to admire people walking.”* (22-year-old woman without any children)

More than a third of women were greatly concerned about their marriages before fistula repair (*n* = 7, 35 %). The relationship status for many women changed after their fistula developed. The majority of women’s marital histories included polygamous relationships (*n* = 15, 75 %) and most of these women became a second wife after their fistula developed (12/15 = 80 %). Additionally, most women attributed their polygamous relationships to fistula. Challenges with intercourse, specifically urine leakage, often stressed their relationships. For those whose marriages remained intact, women often doubted their relationships would continue to survive the challenges of having a fistula.

*“Sometimes I was thinking that this problem will destroy my marriage. Because when I wanted to sleep with my husband it was a problem…I was afraid that with my condition the marriage was going to break up because you know I trusted my husband so much because of the love he was showing to me, but you can’t know what is going on in the heart of your partner. So I was just worried…I was afraid that if this man would have said ‘Leave my house,’ I don’t know where I would have gone. It would have been the end of me.”* (34-year-old woman with 4 living children)

#### Greatest concerns after surgery

Women also shared their current concerns and struggles since having fistula repair. Many of these were different from their concerns before surgery. However, despite undergoing repair, relationship challenges persisted for many women, and some women continued to doubt they would be able to marry. In addition, women were now greatly concerned about: 1) persisting financial challenges, 2) the need for additional surgery, and 3) their fertility and reproductive health since fistula repair.

Although some women did remarry, women continued to struggle in unsupportive relationships after repair. Many women continued to adapt to polygamous relationships and their new role as a second wife, which most described was the decision of their husbands. Four women (20 %) said that having an “unreliable” or “unsupportive” husband was one of their greatest concerns since surgery.

*“I don’t depend on my husband much because he is a polygamist and doesn’t support me much…My biggest worry is about the way my husband behaves. He doesn’t care much about me.”* (51-year-old woman with 4 living children)

Some women feared they would never be able to marry despite undergoing repair surgery. Women attributed their inability to marry to ongoing fistula complications including reduced vaginal size since surgery and the need to delay marriage so vaginal tissues could heal.

*“First of all, I am worried that I am not going to find a man who is going to marry me with the situation I am having now of having a small way [vagina].”* (19-year-old woman without any children)

Women also reported they remained single because it was difficult to find a supportive husband to marry, which was important given their past relationship experiences. For three women (15 %), not having a husband was one of their greatest concerns after surgery.

Interviewer: *You don’t have any other worry?*

35-year-old woman without any children: *Not at all. But maybe again of having a husband. I wish I could have someone whom I can call a husband. Yes. That’s my prayer. That if I go there for the operation God should favor me so that I can have the total healing.*

Despite undergoing repair surgery, sexual dissatisfaction and challenges during sexual intercourse persisted for many women. Additionally, women attributed relationship challenges to these complications with sexual activity. These challenges included pain during intercourse, reduced vaginal width after repair, and fear sexual intercourse could cause fistula recurrence.

21-year-old woman with 3 living children: *The way is too small.*

Interviewer: *Did you try to have sex with him that time?*

21-year-old woman: *Yes, and he could not get through. That was the time before I got fistula. Now things came to worse when I got repaired. The doctor said that I am having a small way [vagina] and I have to come back when I am completely healed so that he can enlarge it.*

Many women were most concerned about the financial challenges that continued after fistula repair (*n* = 8, 40 %). These challenges affected their ability to return to the Fistula Care Centre for their follow-up appointments and surgeries. The timing of another operation also affected some women financially, specifically farmers who would be unable to work during the season.

*“My only worry is about the date which they gave me…Because if they do the surgery I have to stay several months without working and that means this year will pass without farming and the result will be that I will not have food.”* (35-year-old woman without any children)

Another 7 women (35 %) stated one of their greatest concerns was the need to undergo another operation to repair their fistula. Many women with continuing urinary incontinence were frustrated because they doubted that another repair would be successful.

*“My biggest concern now is will the remaining problem be repaired once and for all? Because I want to be dry like my friends. Because some people are saying even if I go for another surgery to finalize the remaining part it will not work. They say I was bewitched and I will remain like this forever.”* (30-year-old woman without any children)

For three women (15 %), their reproductive health and fertility after surgery were among their greatest concerns. Women worried they may be unable to have another pregnancy and delivery. History of hysterectomy, absence of menstruation, and difficulty conceiving contributed to these concerns.

*“Even up to now I still think that if I conceive will I keep the pregnancy to the full term? Because I just think that maybe the child will be too big (like before) for the already weak uterus. I am still not convinced that it can carry the pregnancy…In the real sense I need a child but I am not conceiving.”* (30-year-old woman without any children)

#### Quality of life at the individual level

Compared to their quality of life immediately before surgery, nearly all (*n* = 19, 95 %) women felt the quality of their life had improved after surgery. Even among women who continued to experience urinary incontinence, the improvements in their physical conditions positively influenced the quality of their lives.

*“From the time I was repaired up to now there is a big change in relation to the way I live now. Although there is still a problem but it is not as it was before…I still leak but not like before…”* (27-year-old woman with 2 living children)

The woman, who felt her life had not improved since surgery, continued to suffer from ongoing fistula complications and severe depression.

The majority of women (*n* = 13, 65 %) also reported that the quality of their life after surgery was similar to the quality of life they had even prior to fistula development.

*“The quality of life has been restored back to the way I was in my teens before I developed fistula. Now I can be with my friends without any problem. I don’t have to put on a lot of stuff so that they do not know that I am leaking. The way I used to live before fistula is the way I am living now.”* (30-year-old woman without any children)

During the interviews, women commonly reported feeling a sense of freedom from many burdens of their obstetric fistulas, such as concerns about stigma and social isolation, the financial burden of having to constantly buy soap and clean clothes, and painful blisters due to urine leakage that limited mobility. Additionally, many women shared their now positive outlooks on life, including feelings of relief, hope for their futures, and improved happiness.

*“If you are not happy, there is nothing good which can come out of your life. So with the freedom that I encountered from the repair, I am able to live a happy life.”* (30-year-old woman without any children)

Nearly all women (*n* = 19, 95 %) mentioned achieving confidence and personal growth after their fistula repair, which were attributed to greater independence and relying less on others for support. For some, the educational support they received during their recovery had influenced this confidence in future achievements.

*“I knew from that moment [restarting education at the Fistula Care Centre] that my life would not be the same. I started Form 3, and I have written my exams well. What is remaining is to hear the results…I can see achieving great things in my life with the support I am receiving.”* (30-year-old woman without any children)

Additionally, the majority of women (*n* = 15, 75 %) noted that improved income-earning ability since surgery had positively contributed to their quality of life.

*“I was just worried about how I was going to continue living in that condition. When I wanted to work, I couldn’t. When I wanted to chat with friends, I couldn’t. Even business I could not do…If I would try to sell my products in the market people would not dare to buy from me as long as they discovered that it was me who was selling. But now I can do business freely and the way I want.”* (30-year-old woman without any children)

#### Quality of life at the interpersonal level

At the interpersonal level, women revealed many struggles that had impacted their quality of life before repair. Most women expressed their fistulas had challenged their relationships with family and friends and limited their community involvement. However, reintegrating and strengthening these interpersonal relationships after repair were not challenging for our study population. Women were welcomed back into their communities and most quickly resumed participation in activities.

Because of the smell of urine leakage, women were unable to eat with their friends and family and they were often isolated from these intimate gatherings. The fear of stigma was so strong many women avoided people and went to great lengths to conceal their situation (*n* = 11, 55 %). Some women did not even inform their families about their fistula.

Interviewer: *Were they [relatives] not aware of your situation?*

34-year-old woman with 4 living children: *No…They would have been at the forefront of stigmatizing me.*

Nearly all women had experienced stigma from family, friends, and/or villagers (*n* = 18, 90 %); however, more than half of women (*n* = 11, 55 %) said their relationships had improved since fistula surgery.

*“Before I was repaired my friends or relatives would not like to be close to me. Once they saw me they would run away because of the urine smell. All my in-laws would openly say that I am stinking but now things have completely changed.”* (27-year-old woman with 2 living children)

One woman, who suffered from ongoing fistula complications, continued to experience stigma from friends.

*“It’s just the same as before. Other people they still laugh at me saying that I smell of urine and others they just talk that I am just wasting my time and resources going to the hospital now and then as if there is a solution.”* (76-year-old woman without any children)

Although women had limited community involvement before surgery, nearly all women were able to participate in these social activities after surgery (*n* = 19, 95 %). Women were relieved they could now participate in community construction projects, village banking, and their religious organizations. Half of women also held positions in these groups.

*“I was being stigmatized at church and home and other organizations. For positions I would be sidelined because I was not always available when they were meeting but now I am holding different positions at church and in youth meetings.”* (34-year-old woman with 4 living children)

Additionally, most women (*n* = 17, 65 %), including those with continuing urinary continence, were now able to travel overnight and attend important out-of-town functions like funerals and weddings.

*“I do take part in events which I know that I will not sit down like weddings. So I just move up and down to pass the time so that I should not leak.”* (76-year-old woman without any children)

### Fertility and pregnancy after pepair

In this section of the interview, various themes emerged regarding fertility desire and understanding of current fertility status. We also evaluated pregnancy outcomes and family planning use when applicable.

#### Fertility desires and pregnancy outcomes

The future pregnancy intentions of women varied greatly. The majority of women were concerned that an additional pregnancy could lead to fistula recurrence; however, about half of the women still desired to have another pregnancy (*n* = 9, 45 %). A minority of women had become pregnant since their fistula repairs (*n* = 3, 15 %); two women had miscarried, and one woman was pregnant at the time of the interview.

Some women appeared to have confusion about their fertility and ability to bear children. One quarter of women did not know if their uterus had been removed and some women were very distraught because they knew little about their reproductive health.

Interviewer: *Have you had your uterus removed?*

19-year-old woman without any children: *I don’t have any idea whether they removed it or not.*

Interviewer: *Are you still having menstrual periods?*

19-year-old woman: *I don’t menstruate since the time surgery was done.*

Interviewer: *Why do you think you are not having menstrual periods?*

19-year-old woman: *I just suspect that maybe they removed the uterus, and that is why I am not menstruating.*

At this point in the interview, the woman began to cry.

One woman who expressed confusion about her reproductive health admitted to seeking a traditional healer and taking traditional medicines because she wanted to conceive. She was frustrated because her community continued to harass her about her failure to conceive and her husband was frustrated with the situation.

*“He does complain but not much. Only that when I ask him to buy me a wrapper (chitenje) he will respond with anger that I am asking for a chitenje when he doesn’t have a child. He just tells me to be putting on dresses and a skirt without a chitenje.”* (30-year-old woman without any children)

Feelings of sadness were common among women who desired to have future pregnancies but were unable to become pregnant. For some women, challenges with fertility were interpreted as direct assaults against their ability to fulfill roles as a wife and mother. Problems with fertility were coupled with fears about partner abandonment and concerns that they would never find a supportive husband.

A 27-year-old woman had two children with her husband and was later abandoned because her uterus was removed during repair. She described the following concerns about her inability to bear children and its effects on future relationships:

*“Firstly, I am still young…I stopped giving birth because of this problem of fistula. I can’t even get pregnant because they removed the uterus. So I still have some fears as a woman because each and every woman will need someone who can support her. So if you want to get married to these men of today it’s a problem. As a woman you need a lot of things in life in order to survive but if no one is there to support you it’s when you find out that we end up doing promiscuity which is not good at all…I ask myself, ‘How am I going to keep myself safe?’”* (27-year-old woman with 2 living children)

#### Family planning use

The majority of women were sexually active at the time of the interview (*n* = 14, 70 %); however, only a few women were using contraception and the most common form of contraception was the implant (*n* = 2, 10 %).

One woman was using the implant because she did not think her husband would be supportive during another pregnancy and delivery.

*“I feel like my husband will not be supportive enough to take me to the hospital in time. I was told that once I get pregnant the delivery will be through caesarean section, so because my husband is having another wife I don’t think he will be there for me. For me to be on the safe side. That’s why I had to put in the implant.”* (30-year-old woman with 5 living children)

The other woman was actually recognized by a nurse at a health centre when she was seeking an operation for tubal sterilization. The nurse recognized her as a former fistula patient and strongly encouraged her to return to the fistula hospital for the surgery. Because she did not want her husband to know about another surgery, she chose to use the implant.

### Understanding of fistula

The final portion of the interview focused on the causes of fistula and what can be done to prevent fistula in other women. From this section, the following themes emerged: knowledge of fistula, fistula advocacy to others, and suggestions for helping women with fistula.

#### Knowledge of fistula

In general, women had a fair understanding of the causes of fistula development. They also recognized the steps women should take to prevent fistulas from developing. Half of the women recognized delays in seeking, reaching, and receiving hospital care can contribute to fistula development. Women also described the following as factors that can lead to fistula development: prolonged and obstructed labor; delivering at home or with a traditional birth attendant (TBA); methods used during delivery that damage tissue; families encouraging delivery at the wrong time or place; fear during labor; multiple pregnancies that weaken a woman’s uterus; and witchcraft.

When asked why women develop fistula, nearly half of the women described prolonged and obstructed labor as causes of fistula but many had a poor understanding of the causes of prolonged labor. Moreover, a number of descriptions of prolonged labor were vague. Some women felt that mothers were afraid during delivery and failed to push appropriately, which led to prolonged labor.

*“When someone is failing to push they say it is fear.”* (76-year-old woman without any children)

After fistula repair, a handful of women continued to express their belief that fistulas are due to bewitching practices within their communities. One of these women revealed she was a traditional birth attendant and had helped with deliveries in her village. Similarly, the majority of women revealed people in their communities continue to attribute fistula to witchcraft and fear during delivery (*n* = 12, 60 %).

*“They say that people have done some magic so that when I am going to deliver I should deliver successfully and instead the urine will be coming out all the time. Some believe that the witches took a piece of a reed and closed both sides after putting water in it and threw it back in the water. They say as long as the river will have the running water then urine will not stop.”* (32-year-old woman with one living child)

#### Fistula advocacy to others

At the time of the interview, nearly half of the women knew of other women in their villages who were living with unrepaired obstetric fistulas. The majority of women expressed personal efforts that had helped other women with fistulas and prevented fistula development in other women.

More than half of the women had encouraged other women with fistulas to seek repair and they referred these women to the Fistula Care Centre. Other personal efforts to help women with fistulas included offering to escort women to the Fistula Care Centre, accompanying women during their stay at the centre, and displaying posters that advocated fistula repair.

*“I encourage mothers so that they should know more about fistula. It irked me the time people were saying I have cancer. It forced me to go ahead and tell women what fistula actually is. I tell them the causes of fistula and now they go to the hospital in time.”* (32-year-old woman with one living child)

#### Suggestions for helping women with fistula

Women had a number of suggestions for assisting women with fistula. These suggestions included encouraging women who have fistulas to “come out” or “speak up” about their condition, informing women that fistula repair is available, and developing groups among former fistula patients to mobilize communities, advocate repair, and educate others about fistula.

One woman expressed that personal testimonies from former fistula patients could encourage fistula repair among women. She also noted many women avoid fistula repair because they are advised to abstain from sex following repair.

*“I think they should be encouraged. We have to make sure that they understand thoroughly about this hospital and what a surgery entails. The problem is when we come here and we tell them that once you are repaired you are denied from sex for a period of six months. Now you are telling this to someone who has a husband, that’s why most of them prefer not to be repaired.”* (35-year-old woman with 3 living children)

Another woman, who attributed her partner abandonment to the hysterectomy she received during fistula repair, strongly believed men should be encouraged to support their wives before, during, and after fistula repair. After describing a friend who would not seek repair because her husband did not support her or the procedure, she expressed the following suggestion:

*“I think the messages should not target women only. It should also be directed to them [men] so that they realize that their input is needed and also to let them come back to their senses and know what they are doing is bad.”* (27-year-old woman with 2 living children)

When asked what else could be done after fistula repair, the majority of women suggested providing business loans to patients and more than a quarter of women suggested running a small scale business would help women achieve financial independence after repair. Other common suggestions to help women after repair were providing financial assistance, counseling women on safe motherhood, providing inclusive health education, and providing fistula patients with educational opportunities.

A small number of women also expressed gratitude for the home visit. Some women felt the visit had encouraged them to once again advocate fistula repair to others. One woman also felt the home visit was beneficial because she was in poor health.

*“You should be visiting them the way you have done because it’s like a source of encouragement to women. If you do this we know that there is someone who really cares. You know I didn’t expect this. This shows that our doctors care for us…I think there should be a proper follow up especially for us who still have the problem. There should be a proper linkage between us here and you people there. If you had not come, you would have been unable to know about my condition now. So I think it has to be a routine follow up so you know what is happening to us out here.”* (53-year-old woman without any children)

## Discussion

These qualitative interviews shed light upon the experiences of women in Central Malawi before and after obstetric fistula repair. Despite reported improvements in quality of life, many social, psychological, and economic challenges persisted after fistula repair. For most women, the challenges experienced at the individual and interpersonal levels were different before and after fistula repair, although relationship issues often remained. Financial issues persisted, but were now more similar to other women with the same socioeconomic status and less related to fistula (i.e., they no longer needed to buy as much soap or clean clothes to conceal their incontinence). Fertility intentions ranged widely, depending on whether or not the woman already had living children. Those with living children tended to be afraid of having more pregnancies and disrupting their repair. In contrast, those participants without children greatly desired them, but in some cases, were now unable to have them because of hysterectomy. Understanding of the causes of fistula and strategies to prevent it was generally good, although myths about witchcraft and misunderstanding of the causes of prolonged labor were present. Most women were passionate advocates for fistula repair, and many were educating and assisting others who were in need of repair.

Unlike other studies that found women were not accepted back into their communities after fistula repair [18, 19], the women we interviewed did not report challenges reintegrating into their communities. Our finding reflects previous research by Yeakey et al. that found Malawian women reported few barriers to reintegration after obstetric fistula repair [17]. The fact that most Malawian women do not experience stigma and social challenges during reintegration is a positive outcome of fistula repair surgery. This finding and the narratives of our participants suggest that even among women with continuing urine leakage, fistula repair surgery can positively affect a woman’s quality of life.

Because a number of women were unsure about their reproductive health and fertility, we suggest counseling and educating women about these issues prior to discharge after fistula repair and again at their follow-up visits. Furthermore, few women were using contraceptives despite knowing the risks associated with additional pregnancies after fistula repair, including re-opening of the repaired fistula [20]. A challenging part of the discharge process includes discouraging women from sexual activities for six months after fistula repair, so that the vaginal tissue has ample time to properly heal. But because women are discouraged from sexual activities for six months, they may be less likely to inquire about contraceptive options and availability. This could lead to unintended pregnancies among women who have previously undergone fistula repair. Therefore, we believe a more comprehensive discharge process that includes contraceptive education and a discussion of future fertility goals (if applicable) would be beneficial for women at fistula repair centers.

Nine of the twenty women we interviewed continued to experience varying levels of urinary incontinence 1–2 years after operation(s) to repair their obstetric fistulas. Because this study did not seek to determine the cause of post-repair urinary incontinence (stress, urge, low capacity, or residual fistula), we cannot correlate the cause of incontinence to our findings on quality of life. Since post-repair incontinence is a common phenomenon after fistula repair [18, 21], long-term follow-up of a larger series of patients with determination of the cause of the incontinence is encouraged. Ongoing urinary incontinence among former fistula patients also highlights the importance of follow-up appointments, similar to those conducted at the Fistula Care Centre in Lilongwe, to properly assess the post-operative progress of fistula patients [18].

We are unaware of previous obstetric fistula research that focuses on such long-term outcomes and quality of life after fistula repair. Similar studies have had much shorter follow-up periods and conducted interviews 3–9 months after repair [17–19]. The need to investigate longer-term outcomes after fistula repair has been suggested throughout the literature [17, 18]. Findings from our study further illustrate both the benefits of obstetric fistula repair as well as the ongoing challenges of obstetric fistula that women may endure 1–2 years after repair.

One limitation of the study findings is its generalizability as only women from Central Malawi were interviewed. In addition, eligibility criteria included the ability to locate participants and/or contact them via working phone numbers, and all of our participants had previously returned to the Fistula Care Centre for a follow-up appointment. These criteria and characteristics may not represent the larger population of fistula patients, especially patients from areas other than Central Malawi. A second possible limitation of the study is recall bias concerning questions about quality of life before fistula development; for some women, their fistula occurred many years ago, and they may have forgotten how difficult their lives may have been prior to fistula development. Although our interviewer was associated with the Fistula Care Centre, we do not believe a “courtesy” bias similar to that described by Turan et al. [18] was introduced into the study as our participants were quite willing to discuss the challenges that remained even after fistula repair.

## Conclusions

The findings of this qualitative study revealed that nearly all women in our population, including women with continuing urinary incontinence, had experienced long-term quality of life improvements after fistula repair. Similarly, nearly all women would suggest fistula repair to other women. However, many challenges and concerns remained, and these should be explored further to improve future post-operative follow-up for women. In particular, we need to focus more on addressing relationship counseling, financial challenges, the need for additional surgeries, and reproductive health education. Interventions that focus on these long-term outcomes will provide a more comprehensive approach to fistula care.
